# Cholangioscopy-assisted endoscopic mucosal resection for gallbladder polyp and stone extraction for cholecystocholedocholithiasis.

**DOI:** 10.1055/a-2281-9743

**Published:** 2024-04-03

**Authors:** Wengang Zhang, Ningli Chai, Yujie Feng, Jiafeng Wang, Qingzhen Wu, Zhenyu Liu, Enqiang Linghu

**Affiliations:** 1 Department of Gastroenterology, The First Medical Center of Chinese PLA General Hospital, Beijing, China; 2 104607 Department of Gastroenterology, Chinese PLA General Hospital, Beijing, China

A 58-year-old man with cholecystocholedocholithiasis was assessed in our hospital. Preoperative computerized tomography (CT) showed a 0.6-cm common bile duct (CBD) stone combined with sediment-like gallstones. Therefore, we performed cholangioscopy-assisted extraction through papillary stent 1 for him.

First, biliary intubation was conducted and a single dumbbell-style papillary support was placed in the CBD and papilla. The cholangioscope (eyeMax, 9 F; Micro-Tech, Nanjing, China) was then inserted into the CBD and a black stone was found. A basket was inserted into the CBD through the working channel of cholangioscope and frapped the stone ( Fig. 1 ). We subsequently removed the stone from the CBD by withdrawing the cholangioscope and basket together. The cholangioscope was then inserted into gallbladder through the cystic duct over a guidewire, and the sediment-like gallstones were removed by the aspiration function under direct vision. An approximately 0.2-cm gallbladder polyp was found ( Fig. 2 ). We then performed cholangioscopy-assisted endoscopic mucosal resection (CA-EMR) 2 for the gallbladder polyp using a snare with the electrocision function (Jiangsu Changmei Medtech; Changzhou, China), which can pass through the working channel of a cholangioscope ( Fig. 3 , Fig. 4 ). Finally, naso-gallbladder drainage was performed ( Video 1 ). The patient’s recovery was smooth.

**Fig. 1:**
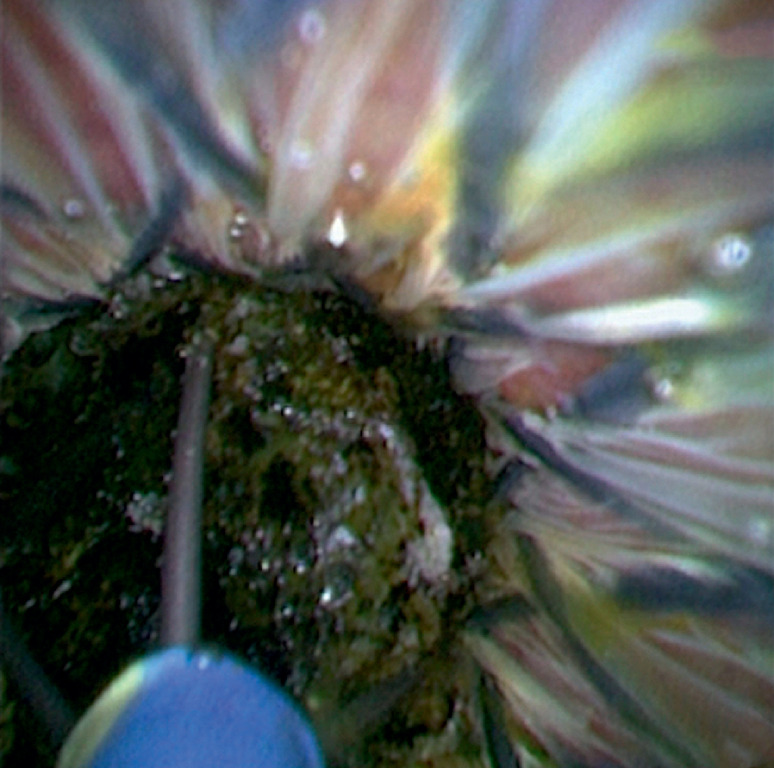
A basket was inserted into the common bile duct (CBD) through the working channel of cholangioscope and frapped the stone firmly under direct vision.

**Fig. 2:**
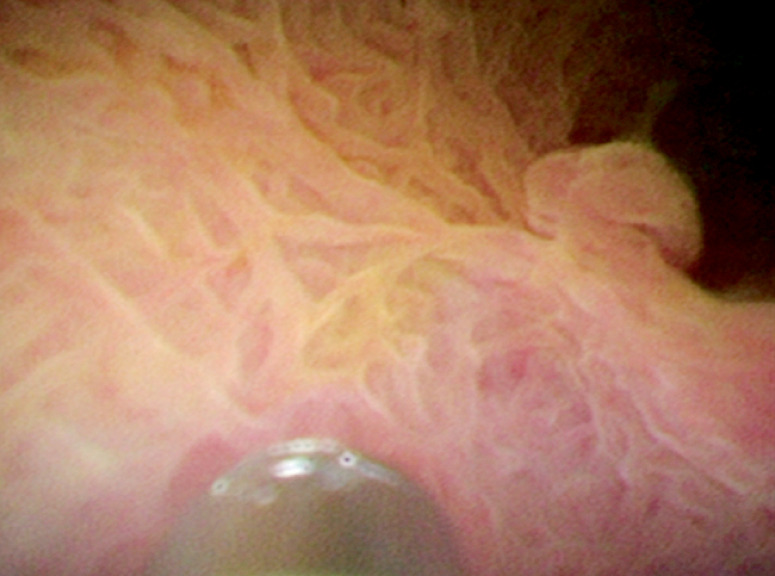
An approximately 0.2-cm gallbladder polyp was found.

**Fig. 3:**
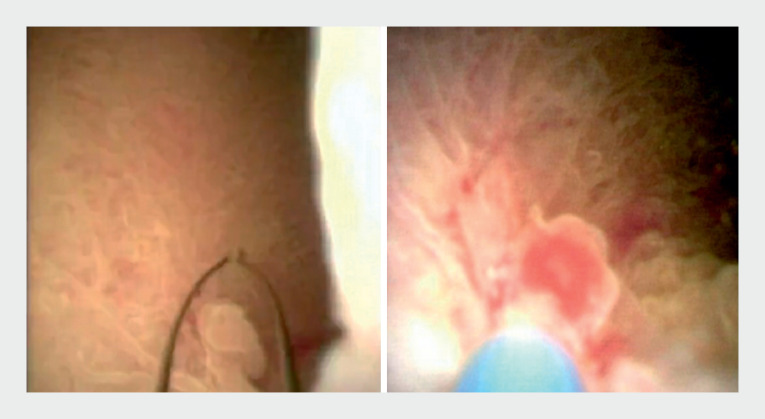
The specially designed snare was inserted into the CBD, and the polyp was resected successfully using the snare by the electrocision function.

**Fig. 4:**
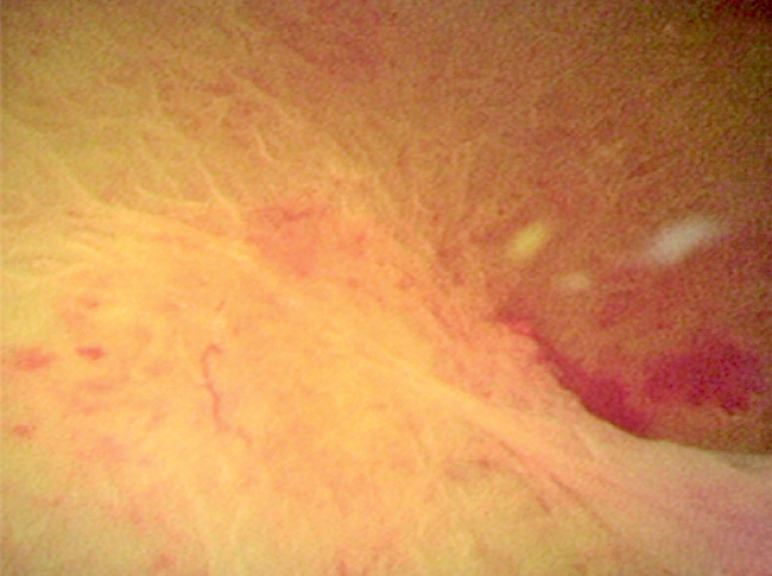
The appearance of the postoperative wound.

The procedures of cholangioscopy-assisted endoscopic mucosal resection for a gallbladder polyp and stone extraction for cholecystocholedocholithiasis.Video 1

With the improvement and popularization of radiological techniques, more and more polypoid lesions in the biliary duct and gallbladder have been found 3 . Patients with polypoid lesions in the biliary system often faced a dilemma. Surgical treatment for polypoid lesions was accompanied by relatively major trauma; on the other hand, follow-up observation came with the risk of progression of the lesions. Recently, our team introduced CA-EMR for CBD mucosa in the porcine model 2 . Subsequently, we successfully performed this technique for a patient with a polypoid lesion in the clinic 4 ( Fig. 5 ). In this study, we further confirmed the feasibility of CA-EMR for a gallbladder polyp in the clinic. Moreover, this study verified that it was feasible to perform cholangioscopy-assisted extraction through papillary stent for CBD stones combined with sediment-like gallstones.

**Fig. 5:**
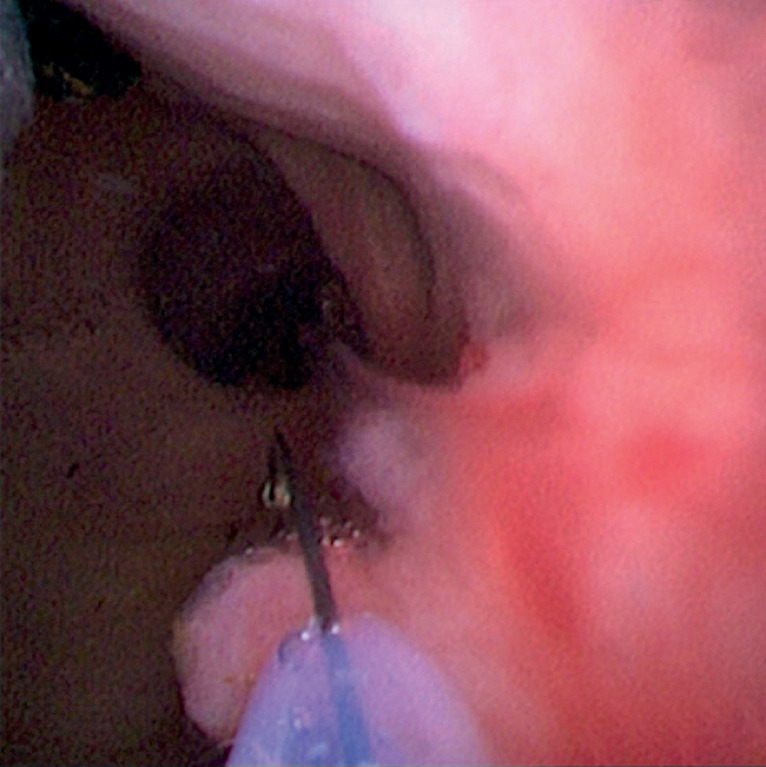
We performed a cholangioscopy-assisted endoscopic mucosal resection for another patient with a CBD polypoid lesion.

Endoscopy_UCTN_Code_TTT_1AS_2AH

## Notes

Endoscopy E-Videos https://eref.thieme.de/e-videos
*E-Videos* is an open access online section of the journal *Endoscopy* , reporting on interesting cases and new techniques in gastroenterological endoscopy. All papers include a high-quality video and are published with a Creative Commons CC-BY license. Endoscopy E-Videos qualify for HINARI discounts and waivers and eligibility is automatically checked during the submission process. We grant 100% waivers to articles whose corresponding authors are based in Group A countries and 50% waivers to those who are based in Group B countries as classified by Research4Life (see: https://www.research4life.org/access/eligibility/ ). This section has its own submission website at https://mc.manuscriptcentral.com/e-videos .
